# MOF–Polymer Mixed Matrix Membranes as Chemical Protective Layers for Solid-Phase Detoxification of Toxic Organophosphates

**DOI:** 10.1021/acsami.2c18691

**Published:** 2023-01-05

**Authors:** Hong-Bin Luo, Fang-Ru Lin, Zhi-Yuan Liu, Ya-Ru Kong, Karam B. Idrees, Yangyang Liu, Yang Zou, Omar K. Farha, Xiao-Ming Ren

**Affiliations:** † State Key Laboratory of Materials-Oriented Chemical Engineering and College of Chemistry and Molecular Engineering, Nanjing Tech University, Nanjing 211816, P. R. China; ‡ Department of Chemistry and Biochemistry, California State University, Los Angeles, 5151 State University Drive, Los Angeles, California 90032-8202, United States; § Department of Chemistry, Northwestern University, 2145 Sheridan Road, Evanston, Illinois 60208-3113, United States; ∥ State Key Laboratory of Coordination Chemistry, Nanjing University, Nanjing 210023, P. R. China

**Keywords:** Zr-MOFs, organophosphorus nerve agents, mixed matrix membranes, catalytic detoxification, solid-state hydrolysis

## Abstract

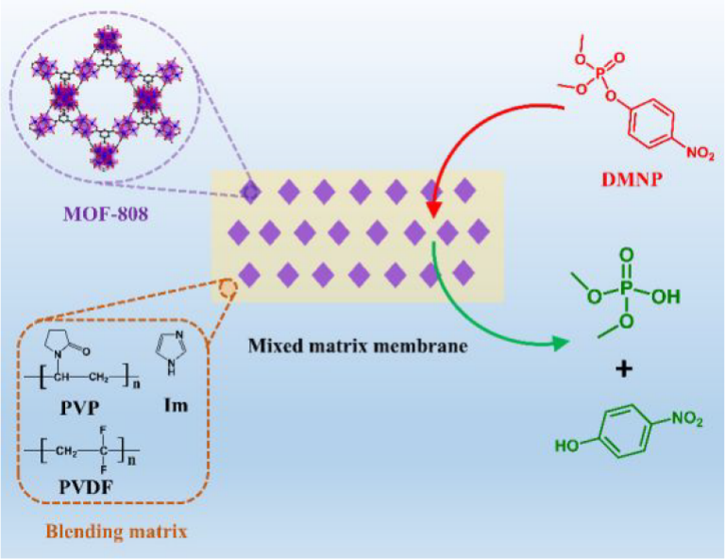

Zirconium-based metal–organic frameworks (Zr-MOFs) have been demonstrated as potent catalysts for the hydrolytic detoxification of organophosphorus nerve agents and their simulants. However, the practical implementation of these Zr-MOFs is limited by the poor processability of their powdered form and the necessity of water media buffered by a volatile liquid base in the catalytic reaction. Herein, we demonstrate the efficient solid-state hydrolysis of a nerve agent simulant (dimethyl-4-nitrophenyl phosphate, DMNP) catalyzed by Zr-MOF-based mixed matrix membranes. The mixed matrix membranes were fabricated by incorporating MOF-808 into the blending matrix of poly(vinylidene fluoride) (PVDF), poly(vinylpyrrolidone) (PVP), and imidazole (Im), in which MOF-808 provides highly active catalytic sites, the hydrophilic PVP helps to retain water for promoting the hydrolytic reaction, and Im serves as a base for catalytic site regeneration. Impressively, the mixed matrix membranes displayed excellent catalytic performance for the solid-state hydrolysis of DMNP under high humidity, representing a significant step toward the practical application of Zr-MOFs in chemical protective layers against nerve agents.

## Introduction

Organophosphorus nerve agents, including sarin, soman, and VX, are a class of the most toxic chemical warfare agents (CWAs) that can easily penetrate human mucosa to disrupt the central nervous system, resulting in constant muscle contraction and even death.^1,2^ The use of organophosphorus nerve agents in terrorist attacks and assassination poses severe threats to human beings.^3^ In this context, there is a growing interest in developing effective materials/catalysts for degrading nerve agent stockpiles and for employment in personal protective equipment.^4−8^

During the past decade, zirconium-based metal–organic frameworks (Zr-MOFs) have been extensively exploited as high-performing catalysts for the destruction of organophosphorus nerve agents and their simulants.^9−18^ Besides the high porosity and remarkable chemical stability, Zr-MOFs are featured by their periodic Lewis-acidic Zr_6_(μ_3_-O)_4_(μ_3_-OH)_4_ clusters that resemble the Zn–OH–Zn active sites of phosphotriesterase (PTE), an enzyme capable of efficiently catalyzing the hydrolysis of organophosphorus compounds.^19−21^ Many Zr-MOFs (*e.g.,* UiO-66, NU-901, NU-1000, NU-1400, MOF-808) exhibited high catalytic activity for the hydrolysis of organophosphorus nerve agents and their simulants in aqueous solutions buffered by *N*-ethylmorpholine (NEM).^9,13,22,23^ NEM is a volatile liquid base to regulate the pH and supply hydroxide ions for regenerating the catalytic sites in Zr-MOFs.^9,14,22^ For example, MOF-808, a Zr-MOF comprised of six-connected Zr_6_(μ_3_-O)_4_(μ_3_-OH)_4_ clusters and benzene-1,3,5-tricarboxylate linkers, achieved instantaneous hydrolysis of nerve agents/simulants in buffered aqueous solution,^23^ representing one of the most efficient Zr-MOF catalysts. Nevertheless, the high volatility of NEM and the requirement of liquid water in the catalytic hydrolysis of nerve agents,^9,24,25^ together with the poor processability of Zr-MOF powders,^26,27^ are major hurdles that limit the practical application of these MOFs in protective layers against CWAs. Hence, considerable efforts have been devoted to addressing these issues to facilitate the practical application of Zr-MOF catalysts in the degradation of nerve agents.

To replace the highly volatile NEM, a series of polymeric organic amines,^25^ such as linear polyethylenimine (PEI), branched PEI, and PEI dendrimers, have been utilized as heterogeneous bases for the catalytic hydrolysis of nerve agents and their simulants. We also recently demonstrated that less-volatile imidazole could be utilized as an alternative base to NEM. By incorporating imidazole molecules into Zr-MOF pores, composites that structurally mimic the active sites of PTE enzyme were obtained, which can rapidly catalyze the hydrolysis of a nerve agent simulant (dimethyl-4-nitrophenyl phosphate, DMNP) in pure water.^24^

The typical powdered form of Zr-MOFs makes them unfeasible for direct application as protective layers. To solve this problem, Zr-MOFs can be integrated with polymeric fibers (*e.g.,* cotton, polyester, polysulfone, and polyamide) to produce MOF/polymer composite catalysts that can be readily processed to afford the desired form for improved practicality.^26−36^ These Zr-MOFs/polymer composites could efficiently catalyze the hydrolysis of nerve agents/simulants in aqueous solutions, but these reactions are typically much slower in the solid phase due to their reliance on water media. The low efficiency of MOF/polymer composites in the solid phase is a major obstacle to their utility as protective layers for CWAs. Very recently, a breakthrough was made by Farha’s group, who integrated polyethylenimine hydrogel with Zr-MOFs on cotton fibers. Their approach gave rise to a series of MOF/hydrogel/fiber composites that achieved fast solid-state hydrolysis of nerve agents/simulants under ambient conditions.^37^ In this design, the polyethylenimine hydrogel serves as the base and, in the meantime, helps to retain water within the microenvironment of Zr-MOFs, enabling the hydrolysis of nerve agents/simulants in the solid phase without liquid water. Still, to this end, very few studies have investigated Zr-MOFs/polymer composites for detoxifying nerve agents in the solid phase, especially in the form of membranes.

**Figure 1:**
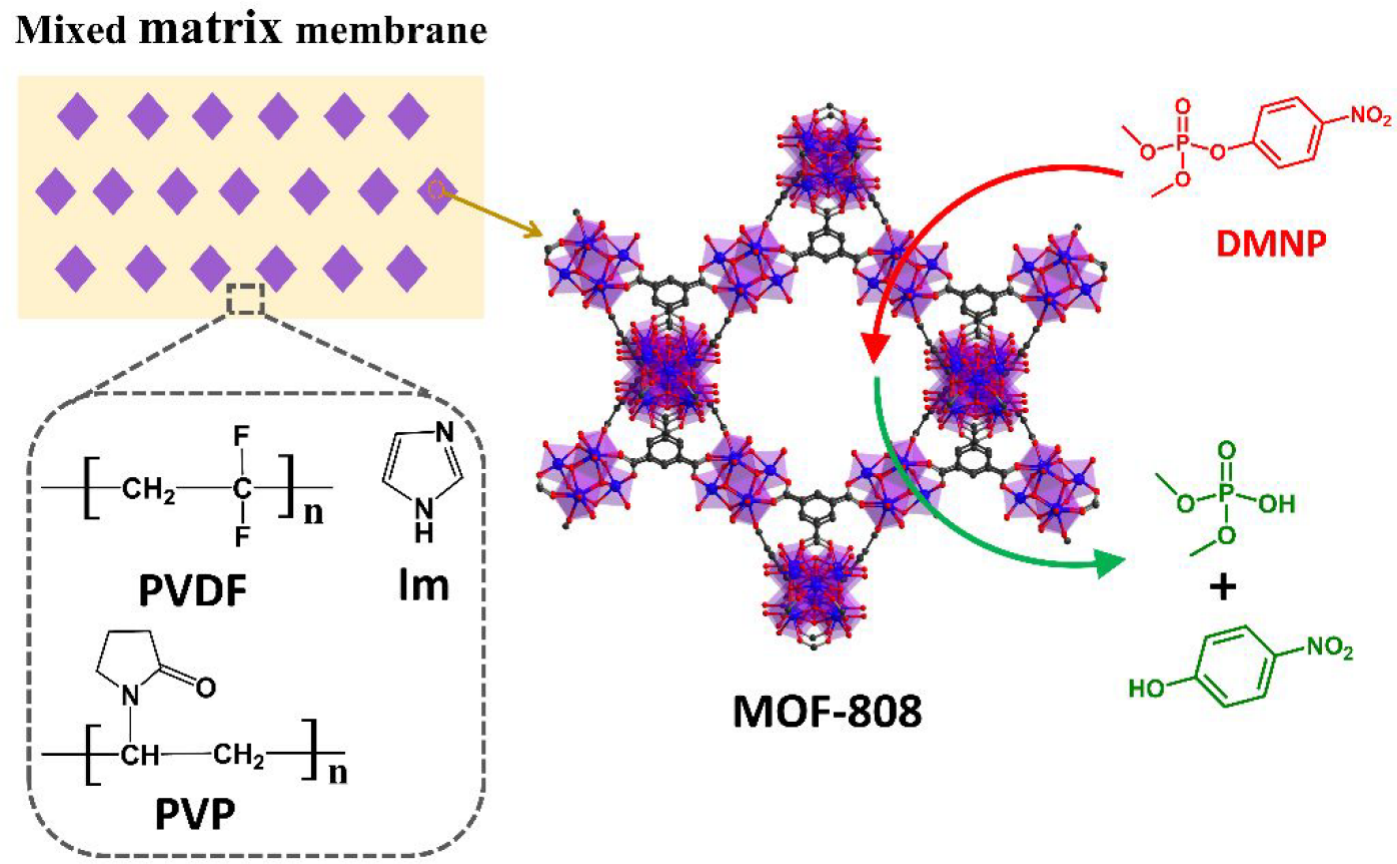
Schematic illustration of the mixed matrix membrane composition used for DMNP detoxification.

Herein, we report the preparation of Zr-MOF/polymer composites by fabricating them into mixed matrix membranes. MOF-808 was selected as a model Zr-MOF in our design due to its exceptional catalytic activity for organophosphate hydrolysis. The mixed matrix membranes were fabricated by incorporating MOF-808 submicrocrystals into the blending matrix of poly(vinylidene fluoride) (PVDF), poly(vinylpyrrolidone) (PVP), and imidazole (Im) (Figure 1). With this design, we anticipated that the resultant membranes would inherit the characteristic properties of each individual component—the outstanding mechanical strength of PVDF will lend the membrane with excellent robustness, the hydrophilic polymer PVP can improve water adsorption/retention of the membranes, and Im serves as an effective base to regulate the pH and regenerate the active sites of Zr-MOFs. Remarkably, our rationally designed membranes showed high catalytic activity for the solid-state hydrolysis of DMNP under high humidity, suggesting their promising application as protective layers against nerve agents.

## Experimental Section

### Materials

All chemicals and reagents, such as zirconium oxychloride octahydrate (ZrOCl_2_·8H_2_O, ≥99.0%, Aladdin), 1,3,5-benzenetricarboxylic acid (H_3_BTC, ≥96.0%, Macklin), *N,N*-dimethylformamide (DMF, ≥99.5%, Macklin), formic acid (CH_2_O_2_, ≥98.0%, Macklin), poly(vinylidene fluoride) (PVDF, Macklin), poly(vinylpyrrolidone) (PVP, Aladdin), imidazole (C_3_H_4_N_2_, ≥99.0%, Macklin), titanium tetrachloride (TiCl_4_, ≥99.0%, Macklin), 4-nitrophenol (C_6_H_5_NO_3_, ≥99.5%, Macklin), dimethyl chlorophosphate ((CH_3_O)_2_P(O)Cl, 96.0%, Aladdin), triethylamine (C_6_H_15_N, ≥99.0%, Aladdin), tetrahydrofuran (C_4_H_8_O, ≥99.9%, Aladdin), ethyl acetate (C_4_H_8_O_2_, ≥99.5%, Aladdin), *n*-hexane (C_6_H_14_, ≥98.0%, Aladdin), acetone (C_3_H_6_O, ≥99.8%, Merckmillipore), magnesium sulfate anhydrous (MgSO_4_, ≥99.9%, Macklin), chloroform-*d* (CDCl_3_, 99.8%, LaboTecc), methyl sulfoxide-*d*_6_ (DMSO-*d*_6_, 99.9%, Macklin) and sulfuric acid-*d*_2_ solution (D_2_SO_4_, 99.5%, Aladdin) were obtained from available commercial sources and used without further purification. DMNP was synthesized by following the reported procedure,^38^ and the details were described in the Supporting Information.

### Preparation of Mixed Matrix Membranes

MOF-808 submicrocrystals were synthesized by following the literature method.^39^ The series of mixed matrix membranes are designated as MOF-808@PP-*X* (*X* = 20, 30, and 40%), where *X* represents the mass percentage of MOF-808 in the membranes. The pure PVDF/PVP membrane is referred to as PP. The mixed matrix membranes were fabricated by incorporating MOF-808 submicrocrystals into the blending matrix of poly(vinylidene fluoride) (PVDF), poly(vinylpyrrolidone) (PVP), and imidazole (Im). The mass ratio of PVDF to PVP was 3:7, and the amount of Im was adjusted according to the amount of MOF-808 in the membrane, with the molar ratio of Im/MOF-808 as 12:1. For example, MOF-808@PP-20% was prepared using the following procedure: MOF-808 (50 mg) was first dispersed in DMF (2 mL) by sonication to afford a suspension solution, followed by the additions of PVDF (52.10 mg), PVP (121.56 mg), and Im (26.34 mg). The mixture was then stirred at room temperature for 6 h to yield a homogeneous slurry, which was then poured onto a glass plate and dried under vacuum at 70 °C for 8 h. Finally, the membrane of MOF-808@PP-20% was obtained by peeling it off from the glass plate.

### Catalytic Hydrolysis of DMNP by Mixed Matrix Membranes

An uncapped vial containing a piece of the membrane (1.0 × 1.0 cm) was incubated in a chamber with relative humidity (RH) of 98% for 24 h. DMNP (12.5 μmol, 2 μL) was added to the vial and dispersed onto the membrane with multiple contact spots. The uncapped vial was then put back into the chamber for different periods of time. Afterward, the reaction mixture was digested in 0.6 mL of 13% (v/v) D_2_SO_4_/DMSO-*d*_6_ and used for ^31^P NMR measurement.

### Characterizations

Powder X-ray diffraction (PXRD) patterns were collected on a Bruker D2 PHASER diffractometer equipped with Cu Kα1 radiation (λ = 1.5406 Å), operated at 30 kV and 10 mA. Thermogravimetric analyses (TGA) were performed using a TA TGA 55 thermogravimetric analyzer under an N_2_ atmosphere. The mechanical properties were investigated on a Shimadzu AGS-X-50N tensile tester. Water-adsorption/desorption isotherms were measured on a Micromeritics 3Flex analyzer. Scanning electron microscopic (SEM) images and energy-dispersive spectroscopy (EDS) mapping were obtained using a Quanta FEG 450 Field Emission Scan Electron Microscope. The air permeability was measured by a PMI Porometer CFP 1500 air permeability meter. NMR spectra were recorded on a 400 MHz Bruker NMR spectrometer.

## Results and Discussion

**Figure 2:**
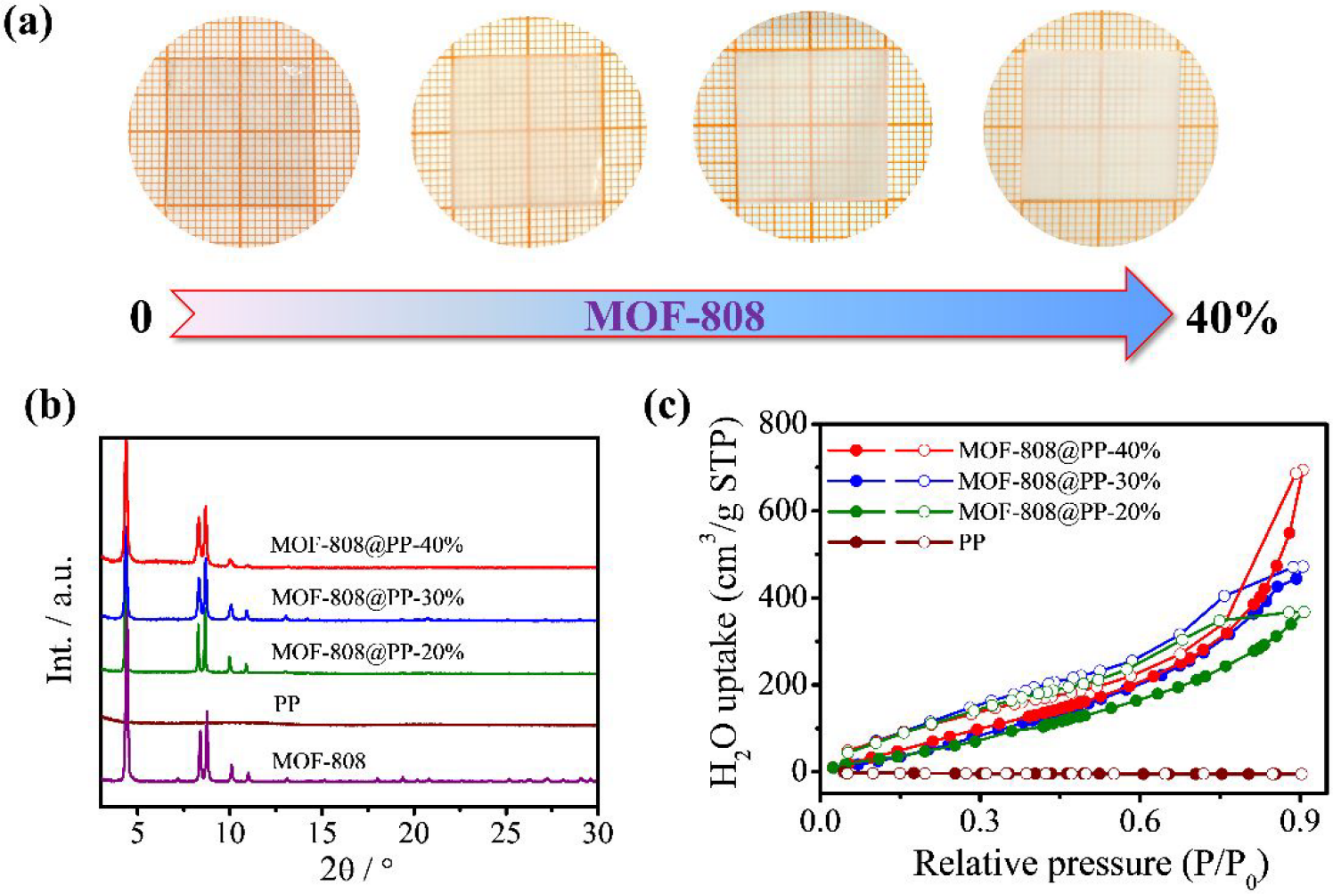
(a) Optical photographs, (b) PXRD patterns, and (c) water-adsorption/desorption isotherms of the pure PVDF/PVP membrane and the mixed matrix membranes of MOF-808@PP-*X* (*X* = 20, 30, and 40%).

**Figure 3:**
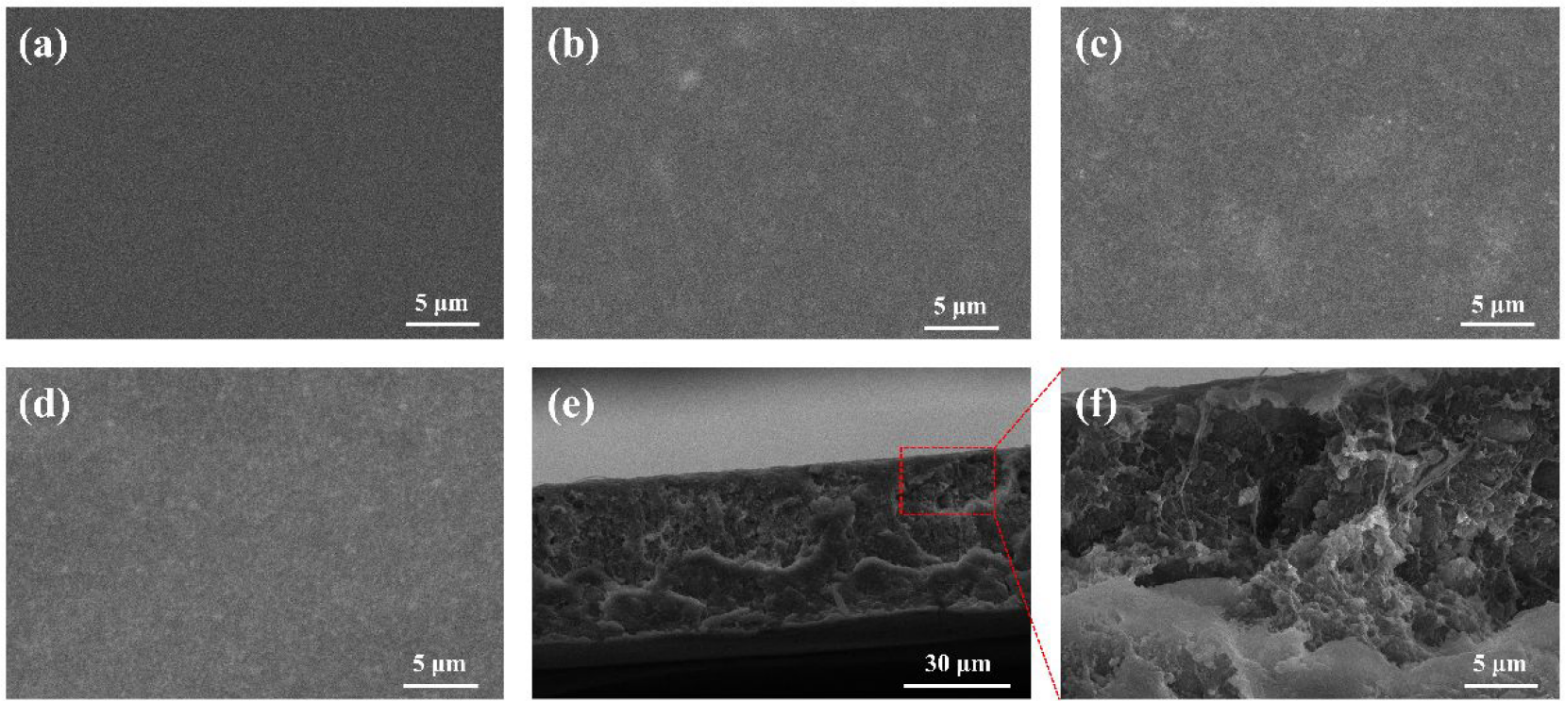
Surface morphology of (a) the pure PVDF/PVP membrane, (b) MOF-808@PP-20%, (c) MOF-808@PP-30%, (d) MOF-808@PP-40%. (e,f) The cross-section of MOF-808@PP-40%.

The mixed matrix membranes were fabricated by integrating MOF-808 submicrocrystals into the blending matrix of PVDF, PVP, and Im. As indicated by the optical photographs in Figure 2a, the membranes showed reduced transparency with increasing MOF-808 content ranging from 0 to 40%. The PXRD patterns of MOF-808@PP-*X* (*X* = 20, 30, and 40%) were compared to the as-synthesized MOF-808, as shown in Figure 2b. In comparison with the pure PVDF/PVP membrane, MOF-808@PP-*X* (*X* = 20, 30, and 40%) showed all the characteristic peaks of MOF-808, suggesting that the structural integrity of MOF-808 was maintained after its incorporation into the supporting matrix. In addition, the surface morphology and cross-section of the membranes were investigated by SEM (Figures 3 and S4–S6). SEM images showed that the membranes had thicknesses in the range of 40–60 μm, and MOF-808 submicrocrystals were well integrated and dispersed in the blending matrix. The homogeneous distribution of MOF-808 submicrocrystals in the membranes was further validated by EDS analysis (Figure S7), which showed uniformly distributed Zr elements in the elemental mapping.

TGA analysis was carried out to assess the thermal stability of the synthesized membranes. As demonstrated in Figure S8, the membranes showed similar weight loss profiles with increasing temperature. The initial gradual weight loss of ∼6.0–8.0% corresponds to the release of adsorbed water molecules from the membranes. The subsequent continuous weight loss starting at 410 K can be attributed to the liberation of Im molecules as well as the decompositions of MOF-808, PVP, and PVDF at elevated temperatures. The TGA data indicated that the membranes were thermally stable up to 410 K. In addition, the stress–strain curves of membranes were measured to evaluate the mechanical properties of the membranes. As shown in Figure S9, the composite membranes of MOF-808@PP-*X* (*X* = 20, 30, and 40%) showed increased elongation relative to the pristine PVDF/PVP membrane, indicating that the incorporated MOF-808 submicrocrystals may serve as a cross-linking agent in the composite membranes. Moreover, the composite membranes can recover from folding and twisting, exhibiting excellent flexibility (Figure S10).

Since water is an essential reactant involved in the catalytic hydrolysis of organophosphorus nerve agents, the water-adsorption capacity of the synthesized membranes was studied. As depicted in Figure 2c, the pure PVDF/PVP membrane showed negligible water adsorption. In contrast, after the incorporation of MOF-808 submicrocrystals, the membranes exhibited a significant improvement in water-adsorption capacity, and their water-adsorption capability improved with the increasing content of MOF-808. MOF-808@PP-40%, with the highest MOF-808 content, displayed the highest water-adsorption capacity among the series of membranes. Essentially, the remarkable water-adsorption capacity of the mixed matrix membranes can be attributed to the incorporation of MOF-808, an excellent water adsorbent featuring high porosity and surface area.^27,40^

**Figure 4:**
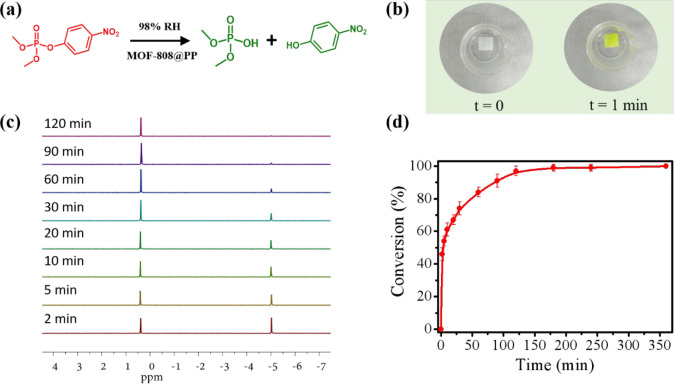
(a) Solid-phase hydrolysis of DMNP catalyzed by MOF-808@PP-*X* (*X* = 20, 30, and 40%) under a highly humid (98% RH) environment. (b) Optical photographs showing the rapid color change of MOF-808@PP-40% membrane (size: 1.0 × 1.0 cm) under 98% RH, resulting from the degradation of DMNP. (c) Representative ^31^P NMR spectra and (d) conversion profile of DMNP hydrolysis catalyzed by MOF-808@PP-40% under 98% RH.

To evaluate the capability of the mixed matrix membranes as chemical protective layers against nerve agents, we studied the hydrolysis of a nerve agent simulant, DMNP, catalyzed by the membranes in the solid phase under high humidity (98% RH) (Figure 4a). As shown in Figure 4b, upon the addition of the colorless liquid DMNP, the membrane of MOF-808@PP-40% showed a rapid color change from pale white to yellow, owing to the generation of the nontoxic hydrolysis product, dimethyl phosphate (DMP). This rapid color change indicated the fast degradation of DMNP by the membranes. To further understand the reaction kinetics, DMNP hydrolysis on the membranes was monitored using ^31^P NMR spectroscopy (Figure 4c), and the conversion was calculated by comparing the integrated ^31^P peak of the substrate DMNP (−5 ppm) with that of the nontoxic product DMP (0.5 ppm). As expected, MOF-808@PP-40%, with the highest MOF-808 content, exhibited the highest catalytic activity toward the hydrolysis of DMNP, achieving 46 and 69% conversions after 2 and 20 min, respectively. Moreover, the hydrolysis profile of DMNP catalyzed by MOF-808@PP-40% presented a short half-life (*t*_1/2_) of 5 min (Figure 4d), featuring it as one of the most efficient MOF/polymer composite catalysts for DMNP hydrolysis.^9,27,36,37^ Additionally, the membrane of MOF-808@PP-40% showed an air permeability of 6.792 × 10^–3^ L cm^–2^ S^–1^, indicating air can pass through the membranes for their practical application as protective layers.

We also investigated the effect of MOF-808 content on the catalytic performance of the mixed matrix membranes for DMNP hydrolysis under 98% RH. The representative ^31^P NMR spectra and hydrolysis profiles are presented in Figures S11 and S12. Compared to MOF-808@PP-40%, the membranes containing less MOF-808 displayed inferior catalytic performance for DMNP hydrolysis. MOF-808@PP-30% converted 37 and 61% DMNP after 10 and 30 min, respectively (*t*_1/2_ = 20 min), and MOF-808@PP-20%, with the lowest MOF-808 content, only converted 19 and 59% DMNP after 10 and 60 min, respectively (*t*_1/2_ = 40 min). In the mixed matrix membranes, MOF-808 is an essential component that provides highly active sites to catalyze the hydrolysis of DMNP. In the meantime, the high MOF-808 content also imparts excellent water-adsorption capacity to the membranes (Figure 2c). Therefore, it is unsurprising that better catalytic performance was observed for membranes with higher MOF-808 content. As a control, PVDF/PVP membranes without MOF-808 were also utilized for the hydrolytic degradation of DMNP, and a fairly low DMNP conversion (22%) was observed after 60 min (Figure S13), placing the PVDF/PVP membrane as the least active (due to the absence of MOF-808). The structural integrity of MOF-808 was maintained in all membranes after catalysis, evidenced by their PXRD patterns (Figure S16).

To date, Zr-MOFs/polymer composites that can achieve efficient solid-state hydrolysis of nerve agents/simulants have been rarely reported.^5,27,37^ The few reported examples employed dip-coating methods to deposit MOFs/polymers on fibers, which may lead to poor mechanical stabilities of the composites because of the weak adhesion between the Zr-MOFs and fibers. Notably, the mixed matrix membranes we developed in this work showed not only high catalytic activity for the solid-state hydrolysis of a nerve agent simulant but also good mechanical properties, rendering them great candidates for practical applications as protective layers against CWAs.

## Conclusion

In conclusion, we presented a viable strategy of incorporating Zr-MOF catalysts into a blending polymeric matrix to fabricate mixed matrix membranes for detoxifying nerve agents in the solid phase. The mixed matrix membranes were prepared through the rational combination of MOF-808 and the polymeric matrix of PVDF, PVP, and Im, in which MOF-808 provides highly active catalytic sites, PVP helps to retain water, and Im serves as a base for regenerating catalytic sites. Remarkably, the rationally designed membranes achieved rapid solid-state hydrolysis of a nerve agent simulant DMNP under high humidity and exhibited excellent mechanical properties. This study represents a significant step toward the practical application of MOF/polymer composites as chemical protective layers against CWAs.

## Supporting Information Available

Details for the synthesis of DMNP, SEM images, NMR spectra, TGA curves, the stress–strain curves, PXRD patterns (PDF)
