# Supplementary material for: MOF–Polymer Mixed Matrix Membranes as Chemical Protective Layers for Solid-Phase Detoxification of Toxic Organophosphates
Source: ACS Appl Mater Interfaces. 2023 Jan 5;15(2):2933–9. doi: 10.1021/acsami.2c18691 (PMC9869327; doi:10.1021/acsami.2c18691)
Supplement: Supplementary file 1 — am2c18691_si_001.pdf [file am2c18691_si_001.pdf]

Supporting Information

MOF-Polymer Mixed Matrix Membranes as Chemical Protective Layers for Solid-Phase Detoxification of Toxic Organophosphates

Hong-Bin Luo,*[a, b] Fang-Ru Lin,[a] Zhi-Yuan Liu,[a] Ya-Ru Kong,[a] Karam B. Idrees,[c] Yangyang Liu,*[b] Yang Zou,[a] Omar K. Farha,[c] Xiao-Ming Ren*[a, d,]

[a] State Key Laboratory of Materials-Oriented Chemical Engineering and College of Chemistry and Molecular Engineering, Nanjing Tech University, Nanjing 211816, P. R. China

[b] Department of Chemistry and Biochemistry, California State University, Los Angeles, 5151 State University Drive, Los Angeles, CA 90032-8202, USA

[c] Department of Chemistry, Northwestern University, 2145 Sheridan Road, Evanston, IL 60208-3113, USA

[d] State Key Laboratory of Coordination Chemistry, Nanjing University, Nanjing 210023, P. R. China

E-mail: hbluo@njtech.edu.cn (HBL); yliu114@calstatela.edu (YL); xmren@njtech.edu.cn (XMR)

Synthesis of dimethyl-4-nitrophenyl phosphate (DMNP)

DMNP was synthesized following the literature procedure (*Chem. Mater.* **2017**, *29*, 2672-2675). Briefly, a solution of titanium tetrachloride (49 μL, 0.44 mmol) in anhydrous tetrahydrofuran (80 mL) was prepared in a Schlenk flask and followed by the addition of 4-nitrophenol (3.4 g, 24.4 mmol). Afterward, a solution of dimethyl chlorophosphate (3.2 g) in 20 mL anhydrous tetrahydrofuran, together with the anhydrous trimethylamine (6.3 mL, 45.3 mmol), was added to obtain a mixture, which was subsequently allowed to stir for 2 h at room temperature under nitrogen atmosphere. The resulting reaction mixture was then quenched with water and extracted with ethyl acetate three times. The combined organic layers were dried over $MgSO_4$, then filtered and concentrated under reduced pressure to yield the pale yellow oily crude product, which was purified by silica gel chromatography with the eluent of ethylacetate/hexanes (1:1). The purity of DMNP was verified by ^1H and ^{13}C NMR spectroscopy (Figure S1 and S2).

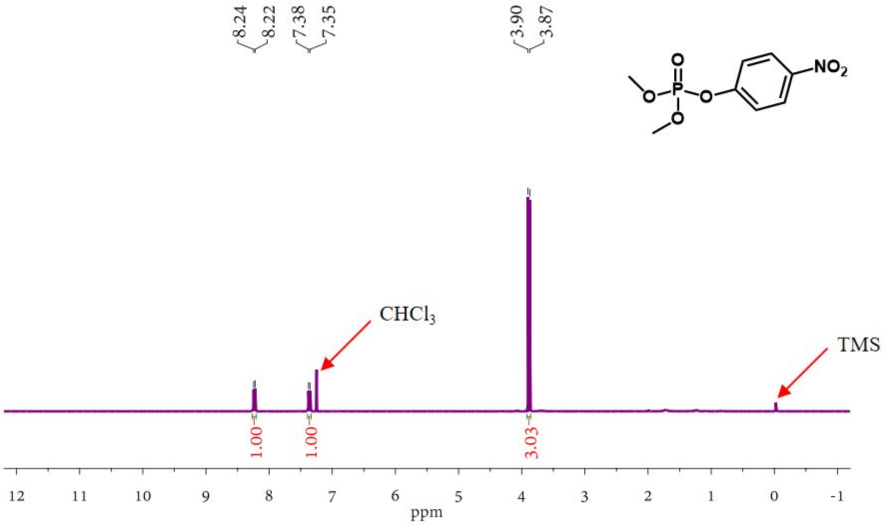

Figure S1. ^1H NMR (400 MHz, $CDCl_3$) spectrum of DMNP.

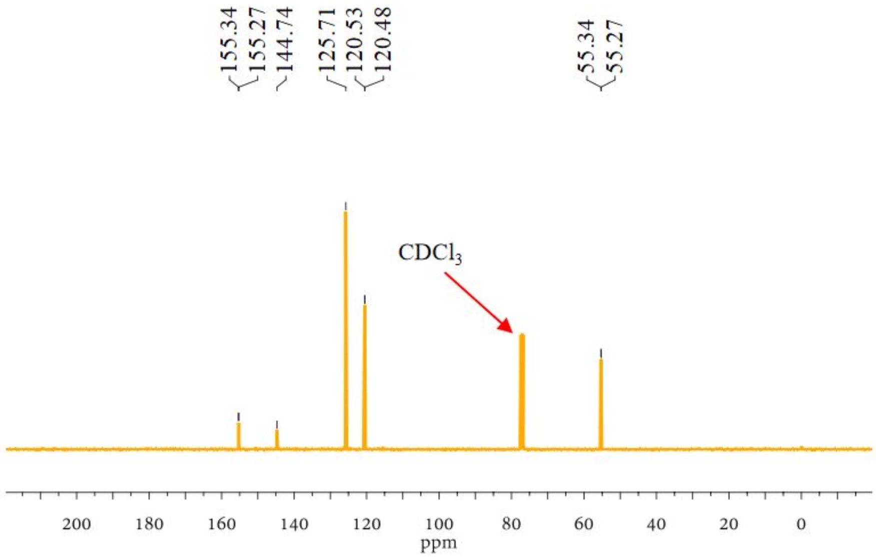

Figure S2. ^{13}C NMR (101 MHz, $CDCl_3$) spectrum of DMNP.

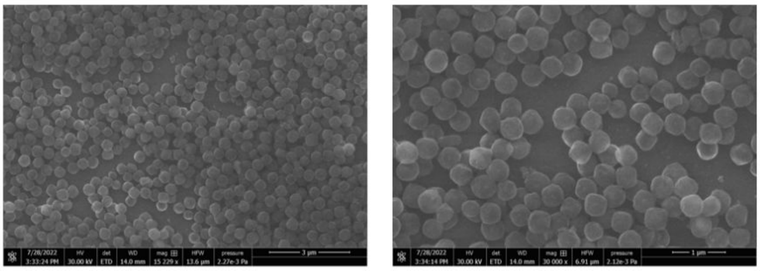

Figure S3. SEM images of MOF-808.

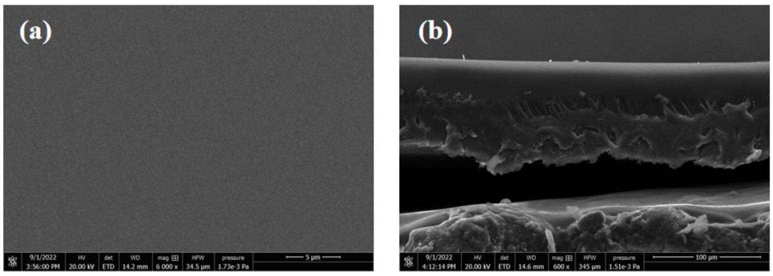

Figure S4. (a) The surface morphology and (b) cross-section of the pure PVDF/PVP membrane.

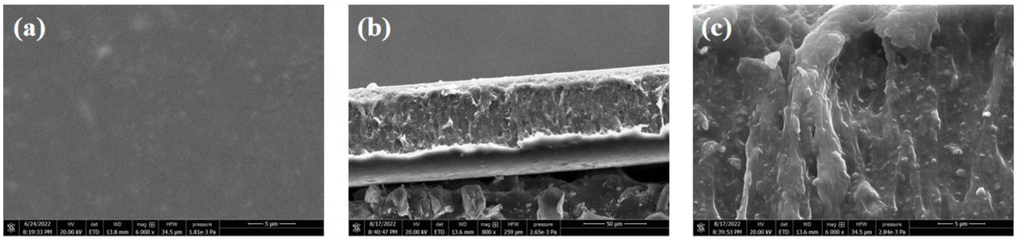

Figure S5. (a) The surface morphology and (b, c) cross-sections of MOF-808@PP-20%.

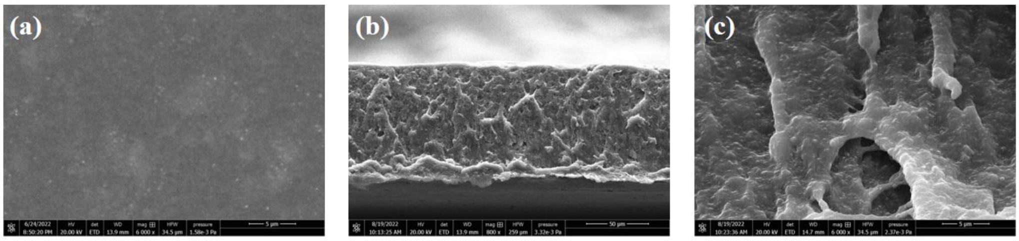

Figure S6. (a) The surface morphology and (b, c) cross sections of MOF-808@PP-30%.

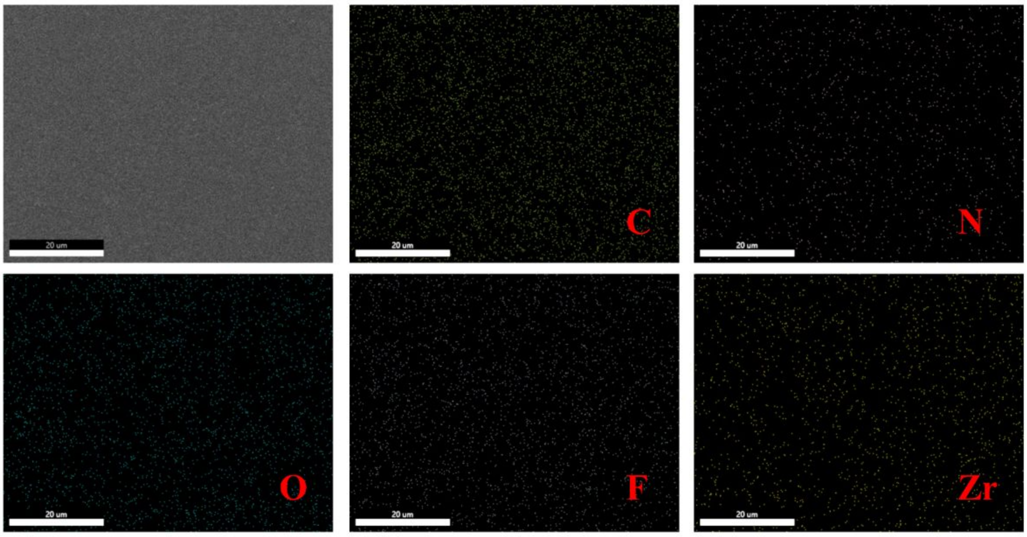

Figure S7. The elemental mapping images of MOF-808@PP-40%.

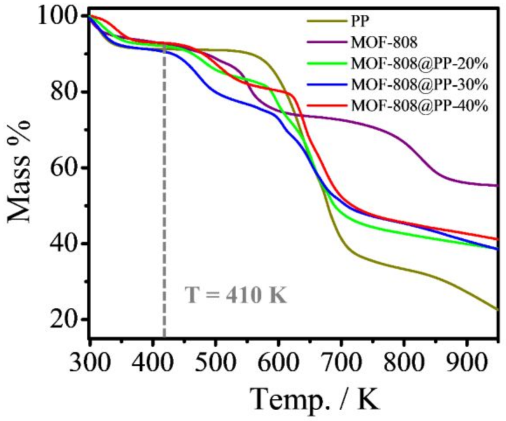

Figure S8. TGA curves of MOF-808, the pure PVDF/PVP membrane, and the mixed matrix membranes of MOF-808@PP-*X* (*X* = 20, 30, and 40%).

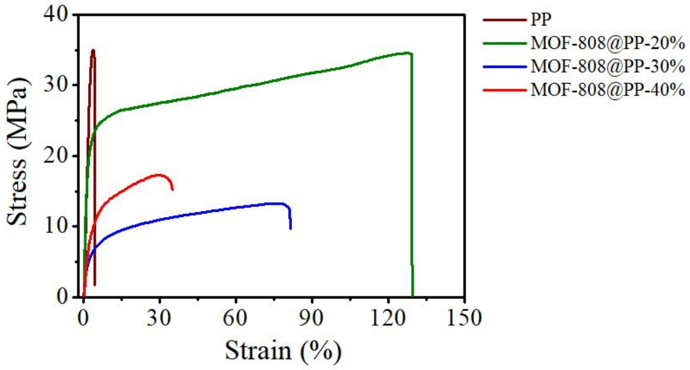

Figure S9. The stress-strain curves of the pure PVDF/PVP membrane and the mixed matrix membranes of MOF-808@PP-*X* (*X* = 20, 30, and 40%).

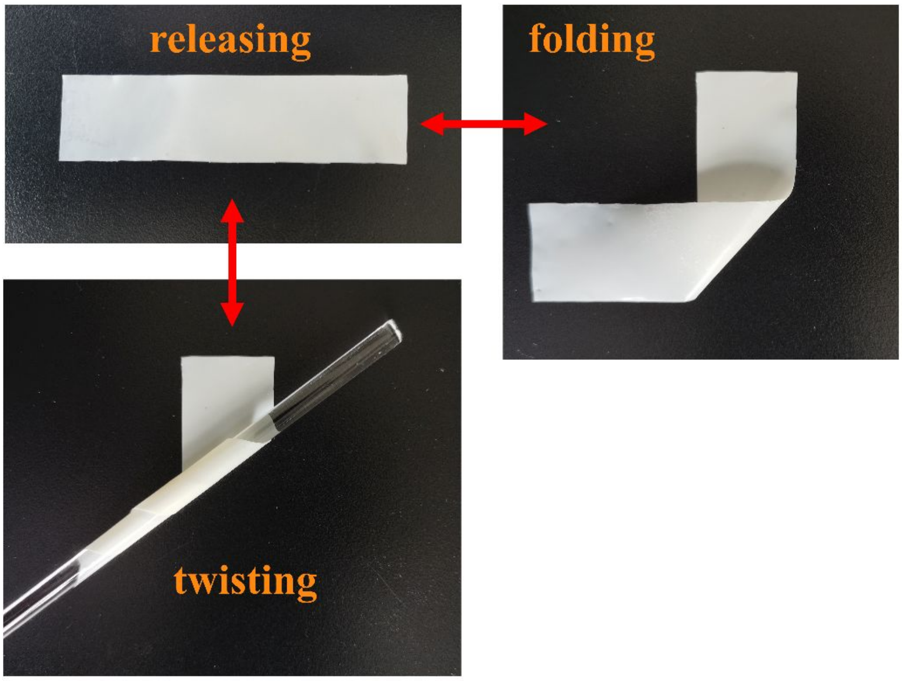

Figure S10. Photographs of folding and twisting of MOF-808@PP-40% (size: 2.0 cm × 8.0 cm).

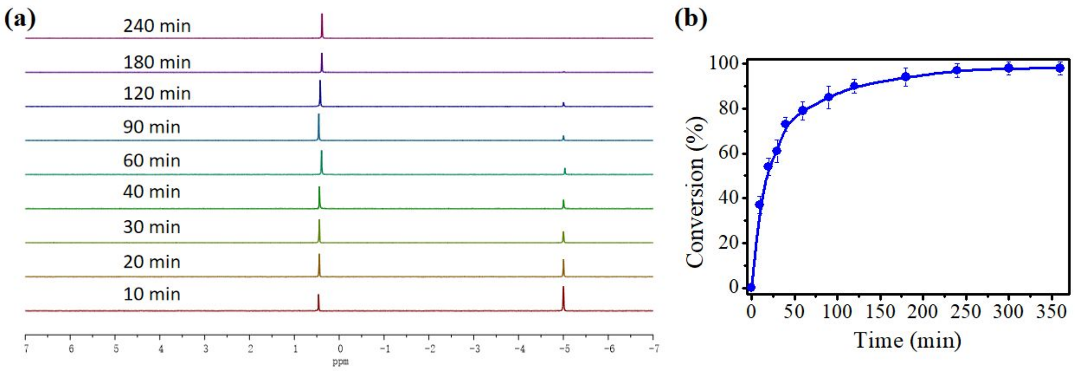

Figure S11. (a) Representative ^{31}P NMR spectra and (b) conversion profile of DMNP hydrolysis catalyzed by MOF-808@PP-30% under 98% RH.

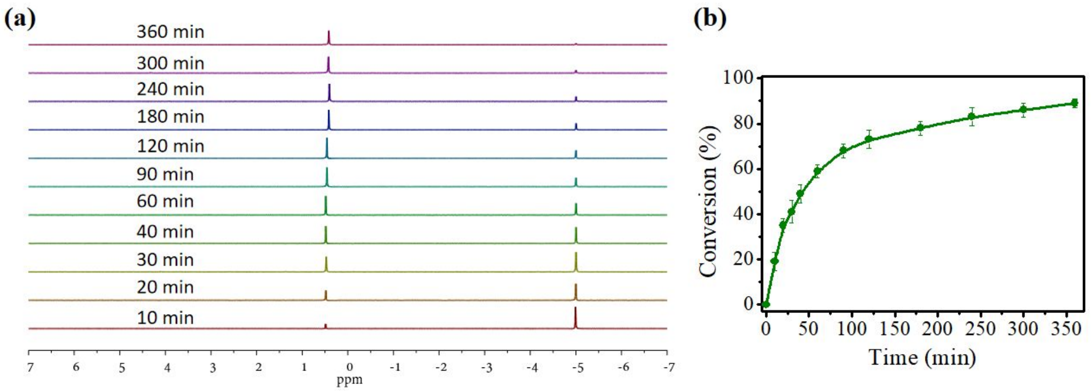

Figure S12. (a) Representative ^{31}P NMR spectra and (b) conversion profile of DMNP hydrolysis catalyzed by MOF-808@PP-20% under 98% RH.

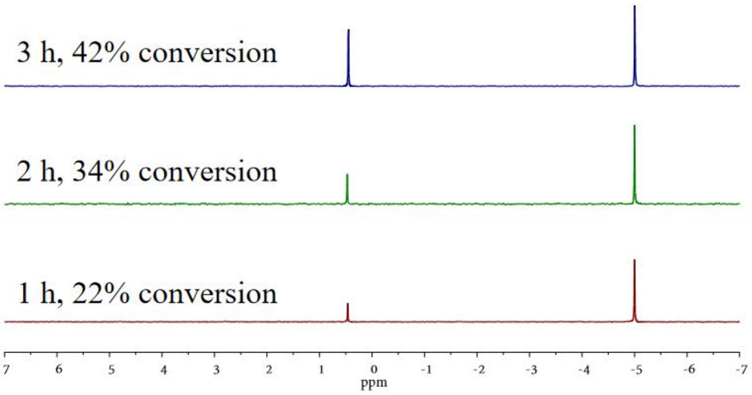

Figure S13. ^{31}P NMR spectra of DMNP hydrolysis catalyzed by pure PVDF/PVP membrane under 98% RH.

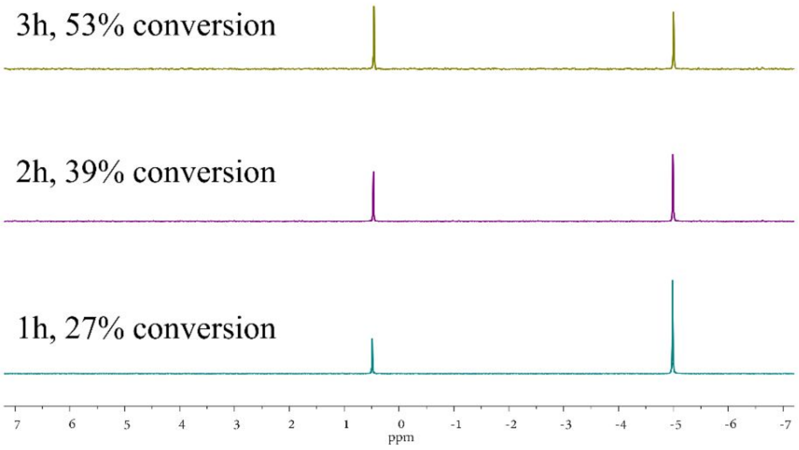

Figure S14. ^{31}P NMR spectra of DMNP hydrolysis catalyzed by PP@MOF-808-40% without Im under 98% RH.

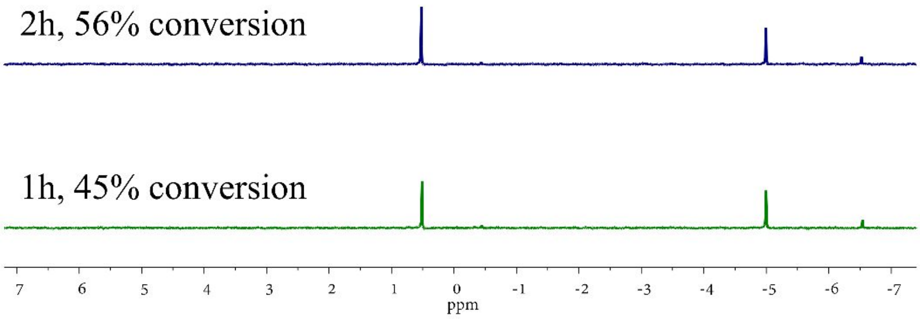

Figure S15. ^{31}P NMR spectra of DMNP hydrolysis catalyzed by PP@MOF-808-40% under 55% RH. The signal peak at -6.7 ppm corresponds to the byproduct of methyl 4-nitrophenyl phosphate (M4NP).[1, 2]

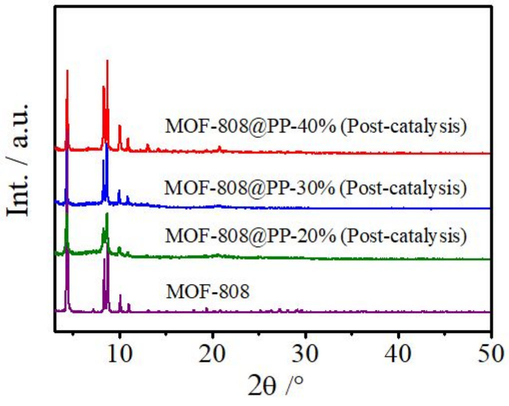

Figure S16. PXRD patterns of the mixed matrix membranes post catalysis.

Table S1. Mechanical properties of membranes

Membranes	Tensile strength (MPa)	Elongation (%)	Young's Modulus (MPa)
PP	35.0	3.8	986.1
MOF-808@PP-20%	34.6	127.7	151.8
MOF-808@PP-30%	13.3	75.7	68.9
MOF-808@PP-40%	17.3	29.8	135.9

References

1. Chen, Z.; Ma, K.; Mahle, J. J.; Wang, H.; Syed, Z. H.; Atilgan, A.; Chen, Y.; Xin, J. H.; Islamoglu, T.; Peterson, G. W.; Farha, O. K., Integration of Metal-Organic Frameworks on Protective Layers for Destruction of Nerve Agents under Relevant Conditions. *J. Am. Chem. Soc.* **2019**, *141*, 20016−20021.
2. Liu, X.; Kirlikovali, K. O.; Chen, Z.; Ma, K.; Idrees, K. B.; Cao, R.; Zhang, X.; Islamoglu, T.; Liu, Y.; Farha, O. K. Small Molecules, Big Effects: Tuning Adsorption and Catalytic Properties of Metal-Organic Frameworks. *Chem. Mater.* **2021**, *33*, 1444−1454.
